# Perceptions of People Living with HIV and HIV Healthcare Providers on Real-Time Measuring and Monitoring of Antiretroviral Adherence Using Ingestible Sensors: A Qualitative Study

**DOI:** 10.1155/2020/1098109

**Published:** 2020-05-27

**Authors:** Susan Kamal, Marc I. Rosen, Christina Lazar, Lisa Siqueiros, Yan Wang, Eric S. Daar, Honghu Liu

**Affiliations:** ^1^University of California, Los Angeles, School of Dentistry, Division of Public Health and Community Dentistry, Los Angeles, CA, USA; ^2^Yale University School of Medicine, Department of Psychiatry, New Haven, CT, USA; ^3^The Lundquist Institute at Harbor-UCLA Medical Center, Los Angeles, CA, USA; ^4^University of California, Los Angeles, Fielding School of Public Health, Department of Biostatistics, Los Angeles, CA, USA; ^5^University of California, Los Angeles, David Geffen School of Medicine, Department of Medicine, Los Angeles, CA, USA

## Abstract

**Objective:**

To describe and analyze the perception and attitudes of people living with HIV (PLWH) and HIV HCPs towards medication adherence with a focus on a digital medicine program (DMP) with ingestible sensors (ISs).

**Methods:**

This is a qualitative analysis pilot study of PLWH who were using DMP recruited by purposive sampling. A convenience sample of HCPs was interviewed. Semistructured interviews were conducted, and thematic analysis was performed.

**Results:**

Fifteen PLWH were interviewed, and thematic analysis resulted in three main themes: self-identified medication adherence patterns, experiences with the DMP, and recommending the DMP to others. Six health care providers (HCPs) described barriers and facilitators to adherence, as well as advantages and disadvantages of using or recommending the DMP to PLWH.

**Conclusion:**

This study evaluated participant and provider responses to DMP, which is a novel technology for real-time measuring and monitoring adherence with the IS. Participant and provider responses were mixed, highlighting both the advantages and limitations of the technology. *Practice Implications*. Taking PLWH experiences into consideration will enhance the development of this and other useful tools that clinicians and researchers can use for enhanced patient care.

## 1. Background

The success of antiretroviral therapy (ART) has made HIV infection a manageable, chronic condition [1]. Although current regimens are more forgiving to occasional missed doses, adherence remains an important predictor of successful virologic suppression [2]. Medication adherence is generally defined as the extent to which the patient follows a medication regimen as intended by the prescriber in collaboration with the patient [3]. Medication adherence has three phases: initiation, which marks the start of the treatment; implementation, which marks the extent to which the patient follows the dosing regimen; and finally, persistence, which marks the continuation of treatment [4]. Nonadherence can occur in any of those phases, such as, noninitiation, premature interruption of treatment, defined as nonpersistence, or suboptimal implementation with isolated or clustered missed doses. ART nonadherence may cause suboptimal clinical outcomes such as an increased viral load and a decreased CD4 cell count [5]. A multicentered study conducted among 768 HIV patients in the U.S., which assessed the relationship between the length of consecutive treatment interruption and increased viral load, showed that viral load starts to increase after 48 hours of consecutive treatment discontinuation [6]. Hence, timely feedback on lapses in medication adherence is necessary to maintain viral suppression.

There are several ways to measure medication adherence, such as pill counts, patient self-reports, pharmacy refill, and electronic monitors [7]. Even though these measures are widely used in clinical practice and research, they are all proxies and infer to actual drug intake behavior. Additionally, they do not actually confirm ingestion of the medication or provide real-time feedback to patients or providers about true adherence. Prior to real-time adherence monitoring, electronic monitoring (e.g., Medication Event Monitoring System (MEMS©)) was considered as highly accurate [7]. However, there are a number of problems with electronic monitoring that make their measurement of adherence suboptimal, such as “pocket-dosing” when patients take several pills out of the pillbox in advance for later use or when the pillbox is opened but pills are not ingested [8, 9]. Other more accurate methods such as drug levels (plasma, urine, and saliva) are expensive and subject to “white-coat” adherence [10]. Real-time monitoring with ingestible sensors of adherence can provide a more reliable alternative to electronic monitors [11–13]. In addition, real-time monitoring provides the possibility for patients to receive distant counseling, for example, when they miss taking their medication. It also prevents extended periods of time with poor adherence which may otherwise go undocumented and unnoticed by the healthcare provider. Furthermore, not all real-time adherence monitoring devices confirm actual pill-ingestions, for example, Wisepill© (an Internet-enabled medication dispenser).

**Figure 1:**
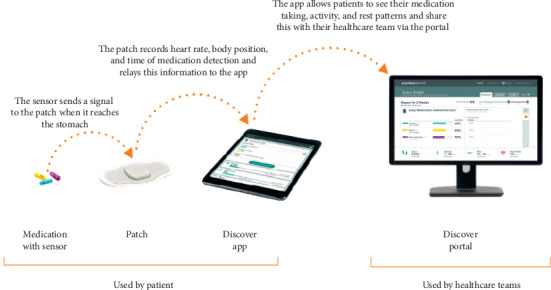
Digital medicine program.

**Figure 2:**
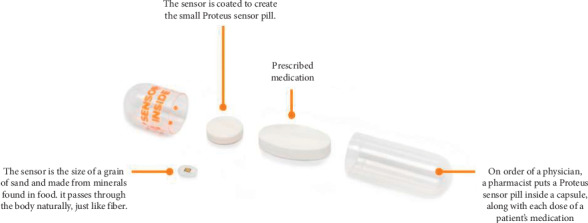
Coencapsulation of antiretrovirals.

A digital medication program (DMP) (Figure 1), which includes an ingestible sensor coencapsulated with medications (Figure 2), a wearable patch, a patient mobile app (iPad), and a provider web portal for real-time assessments of medication ingestion, was recently developed. ARV pills are coencapsulated with an ingestible sensor in each pill. The sensor is activated when the patient swallows the pill and it enters the stomach. Once the capsule dissolves and the stomach fluid reaches the sensor, it then sends a signal to a wearable patch on the individual's body which, in turn, sends the data to a mobile device via Bluetooth. These data are transferred from the device to a secure server that can be accessed by authorized third parties, such as their HCPs, via a web-interface. This allows real-time confirmation of ingestion, which in turn allows for real-time monitoring of adherence and appropriate direction of resources for enhancement of interventions by HCPs and researchers, e.g., SMS reminders in case of a missed dose. The DMP was developed by Proteus Digital Health and has been approved by the FDA. Several peer-reviewed publications have described its safe use and accuracy in measuring adherence in patients with tuberculosis, schizophrenia, and kidney transplantation [8, 14–17]. In our study, Proteus provided technical telephone support in case there were problems encountered with the device use.

In this study, our aim was to describe and analyze the perception and attitudes of PLWH and HIV HCPs towards medication adherence in general and real-time medication adherence with a focus on the DMP.

## 2. Methods

PLWH were recruited as part of an open-label pilot study preceding an ongoing clinical trial (trial registration number is https://clinicaltrials.gov/ct2/show/NCT02797262 for measuring and monitoring adherence to ART with the DMP between February and September 2017. The aim of the pilot study was to determine the acceptability and feasibility regarding the use of the DMP before the trial started among PLWHIV [18]. HCPs were recruited and interviewed in June-July 2019. The initial results were shared in the 12th International Conference on HIV Treatment and Prevention Adherence (IAPAC) in June 2017 [19], and the results confirmed that the drug levels of six different coencapsulated ARVs were consistent with historical values. The ongoing clinical trial has a bigger sample size (*n* = 120) and longer follow-up (28 weeks) than the pilot study.

### 2.1. Setting, Recruitment, Inclusion Criteria, and Data Collection

The recruitment of PLWH who participated in the pilot DMP study and HIV HCPs was conducted at the Los Angeles Biomedical Research Institute at Harbor-UCLA Medical Center, a research institute of a safety net hospital in Los Angeles, California, via purposive sampling [20]. The inclusion criteria were as follows: HIV-infected individuals in HIV care; greater than 17 years of age; able to take coencapsulated ARVs at the time of screening; able to provide informed consent; on ART with current or at an increased risk of suboptimal adherence estimated by either the patient (self-reports < 90% adherence over last 28 days by asking patients how many doses were missed) or treating HCP perception (e.g., based on missed clinic visits or viral load elevations (viral load >200 copies/mL) within the last 6 months). Patient follow-up was per standard of care, in clinic, according to the US Department of Health and Human Services (DHHS) guidelines [21]. Participants in the pilot DMP study were approached about the study and, if willing, provided full study disclosure and methodology, as well as provided informed consent for qualitative interviews, audio taping, and analysis of the information. Semistructured telephone interviews were conducted three days after beginning use of the system and again at week two and at the first of the monthly face-to-face data collection visits for the DMP pilot study. The interviews were all conducted by the study coordinator (L. S.), who was trained in qualitative interviewing and was provided with a semistructured interview guide (Appendix I-a). The interview guide referred to this adherence measuring and monitoring system as the Ingestion Sensory System but is referred to here as the DMP.

Each patient was paid $50 compensation for each DMP pilot study visit (of the three interviews included in the qualitative study, one was conducted during a face-to-face DMP study visit and was compensated, and the rest were conducted over the phone).

HIV HCPs working in the clinic where the DMP pilot study was conducted were recruited through convenience sampling for enrollment in a qualitative interview study by sending them informational emails about the study and follow-up reminder emails to nonresponders. Some of the HIV HCPs cared for PLWH enrolled in the DMP pilot study, and others did not have any PLWH enrolled in the study. The interviews were conducted by phone, and all participants agreed to audio-record the interviews. HCPs were interviewed using semistructured interviews using a semistructured interview guide (Appendix I-b), by the first author (S. K.) who is a pharmacist and a trained qualitative researcher. HCPs were not remunerated for participation.

### 2.2. Data Analysis

Demographic and clinical characteristics of participants including PLWH and HIV HCPs were described using frequencies and percentages or median and interquartile ranges as appropriate. All analyses were completed using the R statistical package, version 3.3, and RStudio version 1.0.136 (R, a language and environment for statistical computing, R Foundation for Statistical Computing, Vienna, Austria (URL: http://www.R-project.org)). All interviews were transcribed verbatim by the computer-assisted qualitative data analysis package “QSR NVIVO version 10” [22]. Content analysis was conducted to identify patterns and commonalities in the data [23]. The coding was done by two independent raters (S. K. and C. L.). The first rater (S. K.) identified and grouped the different themes together, and then the other rater (C. L.) discussed them. There was consensus between the two raters on the themes identified, which strengthens the reliability of the analysis.

### 2.3. Ethical Review

This study was approved by the UCLA-Harbor Institutional Review Board (IRB) committee (IRB number 30621-01, approved on 06/07/2017) and UCLA IRB committee (IRB number IRB#19-000910, approved on 6/13/2019). All collected data and information were stored on a password-protected computer and accessed only by the researchers. Full names of participants including PLWH and HIV HCPs were not recorded; they were assigned codes instead to ensure their anonymity.

## 3. Results

Fifteen PLWH were included. One declined audio-recording. Baseline characteristics are presented in Table 1. Six HCPs were included and are described in Table 2. The thematic analysis resulted in themes pertaining to the following broad topics: self-identified patient medication adherence patterns; experiences with the DMP system including the patch, the pill, the text messages, and the tablet; opinions on the DMP system technical support; recommendations for improving the system for patient interviews. The main themes (code categories) from the HCP interviews were as follows: barriers and facilitators to antiretroviral adherence; advantages and disadvantages of the DMP; and recommending the DMP. In the following sections, we discuss the themes in more detail.

## 4. Section I: PLWH

### 4.1. Self-Identified Medication Adherence Patterns

PLWH described several self-management techniques when asked how they usually took their medication and how they remembered to take it. For example, six participants described taking the medication at a specific time every day: *“I pretty much take the medication approximately within 2 hours of waking up in the morning around 8 am to 9 am”* (P8, male, black, 51, and detectable)*, “I take it with food around 7 o'clock”* (P7, male, black, 51, and undetectable). One participant described keeping the medication in a certain location, to help them remember: *“I keep it on the night stand next to me in the bed, so it's really the first, first, thing I do when I wake up”* (P11, male, Latino, 44, and undetectable). Two described using alarms: *“Well I have my alarm set on my phone every night at 7pm. So, when it goes off I either take it with a piece of fruit or something. For when I am not home, I make sure to take it when I get home immediately”* (P1, male, black, 51, and undetectable). One described the aid of pillboxes: *“I put the medication in weekly little boxes Monday to Sunday and that's how I'm remembering to take it and I have all my meds in one little pillbox like they're all divided each day. Everything's just together and that way I don't have to go through all the bottles”* (P14, male, white, 57, and undetectable).

When PLWH were asked how they remember to get their medication from the pharmacy, for example, one described getting a call to pick it up: *“My pharmacist calls me up every month on the phone or even message that the medication is ready to pick it up and I go pick it up immediately, I don't wait a day or two”* (P2, male, Latino, 58, and detectable). Participants were asked about times when they changed their medication-taking routine and how they adapted their medication adherence behavior. One responded as follows: *“I only change it when I go to my cousin's house to spend the night with them or my sister's house. So I'll have a little container that contains my evening and morning medication”* (P2, male, Latino, 58, and detectable).

### 4.2. Experiences with the DMP

PLWH were asked to describe their experiences using the DMP at day 3, week 2, and week 4 of the 16-week DMP pilot study. They were asked about specific aspects of the DMP including the coencapsulated pill, the patch, getting text messages, and using the tablet.

### 4.3. Coencapsulated Pill

PLWH were asked about using the coencapsulated pills with the sensor in them. Five participants found it easy to take: *“It's not difficult, if anything it's easier because of the coating of the capsule, it's not too big, I just take the pill, I swallow with water or coffee, it's pretty simple, easy not too hard to digest”* (P8, male, black, 53, detectable). Another said as follows: *“It's just a little bigger than usual because I'm used to taking, but it's probably no bigger than an 800 mg Ibuprofen, so I don't have a problem with it”* (P11, male, Latino, 44, and undetectable). Others had a different opinion: *“They are over-sized. I guess you can make them smaller to be more swallow-able”* (P7, male, black, 51, and undetectable).

### 4.4. Patch

PLWH were asked about wearing the DMP patch. Seven participants found it inconvenient, for example, P7 (male, black, 51, and undetectable), *“Sometimes it itches. Sometimes when I sweat it won't stick, then it falls off then I have to change the patch. I wanted to make sure it stuck to my skin so I might have pushed it too hard, so if I take it off it kind of hurts. It is kind of inconvenient to me when I take a shower”* and P15 (male, black, 53, and undetectable), *“Well it keeps frustrating me because it's been quite hot and I've been sweating and most of the time it's about problems keeping it patched on*.*”* Others have adapted to it over time: *“At first I had to get used to it, but I'm used to it now. And I put a piece of like medical adhesive tape over it, so it won't fall off and make sure it stays on. The heat loosens it.”* (P14, male, white, 57, and undetectable). Others reported no problems: *“It is comfortable, it has not given me any side effects of any sort, it is working well”* (P2, male, Latino, 58, and detectable).

### 4.5. Text-Messaging

When asked about getting text messages, five PLWH found them helpful: *“They're good because they remind me, I didn't take my pills and it's a good reminder”* (P7, male, black, 51, and undetectable). On the other hand, three did not like it: “*They send the text like don't forget don't forget don't forget and it was like nerve wrecking, it just kept going on and on.*” (P8, male, black, 53, and detectable) and *“I did get text messages saying to ‘now take your medication' but I already took it”* (P14, male, white, 57, and undetectable).

### 4.6. Smart Tablet/iPad

PLWH were given a tablet for use during the study as part of the DMP. Two participants had technical difficulties with using the tablet, for example, P15 (male, black, 53, undetectable), *“it was just one time when the tablet I was provided with wasn't responding it was all black so I had to turn it off and let it reboot and in about 10 min it was fine.”* and P6 *(male, black, 51, detectable)* who took the pill but was not close to the tablet, so it was not immediately registered: *“I worried a lot about the tablet because sometimes it says I didn't take my meds but then I knew about keeping it in my pocket or near my body.”* Others seemed to like the features of the tablet: *“It tells me how many steps I took and my heart rate, which I enjoy”* (P11, male, Latino, 44, and undetectable).

### 4.7. Experience with DMP-Related Technical Support

PLWH were asked to describe the communication with the DMP technical team when they needed it. Those who had contacted them described the communication as follows: “*It was good. Communication was simple. I wasn't with the iPad at that time so we set up another time and that was great as there were some problems but they (Proteus Call Center) were courteous and friendly*” (P8, male, black, 53, and detectable). *“Yeah. They were all very helpful. Very, very helpful. And patient. So, I'd give them a ten on that”* (P13, female, Latina, 59, and undetectable).

### 4.8. Overall Experience with the DMP

When asked to describe their overall experience with the DMP, six participants reported liking it: *“It's been cool, really interesting. The iPad works, the capsules work, it works! What I really like about this system it monitors your heart, so this really helps me, it also monitors my steps, and how far how long I laid down”* (P1, male, black, 51, and undetectable). Two revealed that it helped them with their medication-taking: *“It's been good, I'm used to it. It teaches me a point where taking my medicine at the right time. I believe taking it at the same time a day is really important”* (P14, male, white, 57, and undetectable). Others reported using the system was inconvenient*: “It's been a new kind of responsibility, as far as having a reminder of the medication and wearing the patch and to follow to replace it constantly, so it's kind of like babysitting myself”* (P5, male, Latino, 49, and undetectable) and *“It's been inconvenient because I didn't have this problem before, that somebody is watching, for me it is kind of inconvenient”* (P3, male, black, 55, and undetectable).

### 4.9. Recommending the DMP to Others

PLWH were asked if they would recommend the DMP to others and why. All who offered an opinion recommended it: *“They should do it if they want to help maintain the practice of taking their meds. They should be taking them at certain times”* (P7, male, black, 51, and undetectable). *“If they are having trouble taking their medication, I'd advise them to participate”* (P3, male, black, 55, and undetectable). *“I would reassure them that it's worth the while, it's very interesting, it's not hard to use, it does everything itself, it's something that will benefit not only ourselves but other people”* (P5, male, Latino, 49, and undetectable).

## 5. Section II: HIV Healthcare Providers

### 5.1. Barriers and Facilitators to Antiretroviral Adherence

HCPs described the barriers that their patients faced with ARV adherence. These included substance use, mental health issues, financial issues, homelessness, unacceptance of HIV diagnosis, and forgetfulness. Factors that facilitated ARV adherence included trusting their HCP, patient motivation and taking responsibility of their health, patients' perceived health benefit, medications that are easy to take with simple one-pill-a-day regimens, proactive HCPs that remind patients about their clinic appointments, social and psychological support (from family and friends), and routinizing the patient's pill-taking behavior, for example, taking the medication every day with breakfast or at bedtime. They also mentioned the use of aids to help with adherence such as alarms, pill organizers, and pill packs.

### 5.2. Advantages and Disadvantages of the DMP

All HCPs knew of the DMP prior to the interview, as the DMP pilot study was conducted at their workplace. Five of six were treating patients in the DMP study. HCPs also seemed to understand how it works. When asked how the DMP helped their patient with ARV adherence, they mentioned that the text reminders are the most important aspect in addition to the fact that the patients feel monitored by their HCPs, which can motivate them to remember to take their medication. On the other hand, when asked about potential difficulties with the system, providers mentioned that the patch can be uncomfortable and that some patients can find it stigmatizing. Furthermore, the availability of a stable Internet connection needed to operate the system can be impossible for some patients with socioeconomic difficulties who are unable to purchase wireless Internet services. When asked if their patients had used other electronic monitors in the past, some mentioned using Wisepill© and MEMS©. They explained that the DMP would be better in measuring adherence compared to MEMS© as some patients opened the pillbox without ingesting their pills, which gave an inaccurate measure of adherence. For the Wisepill©, HCPs complained that the box was too big and there were no text message reminders.

### 5.3. Recommending the DMP

When asked if they would recommend the DMP, HCPs said that they would recommend it to patients who have difficulties with adherence for short-term use, up to 6 months. One HCP puts it as follows: *“For those patients with adherence difficulties, there isn't much left to offer, we tried everything, social work, patient navigators, reminders, none of it was a success. I definitely recommend the IS system”* (H4). They also mentioned that for patients included in the study, there was a financial compensation to use the DMP and that perhaps patients would be less motivated to continue to use it once they no longer receive compensation.

## 6. Discussion and Conclusion

### 6.1. Discussion

In this study, we describe and analyze PLWH experiences and HIV HCPs' opinions on real-time adherence monitoring with a focus on the DMP. The views of PLWH and HCPs were very similar. They only differed on the in-depth explanation of facilitators and barriers to ARV adherence, where HCPs provided more comprehensive reasons on why their patients were not adherent.

Both PLWH and HCPs agreed that DMP can be helpful in the management of ARV adherence. This was similarly reported by persons living with schizophrenia and their HCPs [24] and PLWH who used other real-time ARV adherence monitoring devices such as Med-e-Monitor and Wisepill© [25, 26]. Indeed, there are several advantages of real-time adherence monitoring, as reported by our PLWH who used the DMP and the HCPs. Not only does real-time adherence monitoring provide a reminder, but it also helps patients change their behavior as they become aware of their own adherence patterns and try to mitigate nonadherence in nonroutine circumstances such as being away from home.

The interviews showed that PLWH had already developed self-management tools for medication-taking before using the DMP such as storing their medication at a specific location, taking their medication at the same time every day, or using alarm clocks. Despite that, PLWH were selected for this pilot study because they were having suboptimal adherence (signified by missed doses and/or detectable viral load) prior to their inclusion in the DMP pilot study. This meant that there was still a need for a tool that would further enhance their adherence. By combining self-management with immediate intervention in case of a lapse in adherence, DMP may provide greater support to patients with inadequate adherence. Some patients found this useful and could incorporate it into their daily routines. Similar experiences were reported for patients with schizophrenia and hypertension who used the DMP [24, 27]. In contrast, others found the DMP too demanding, to the extent of being “nerve-wrecking,” and mentioned that it takes getting used to. This confirms that adherence is a very individual behavior and that there is no one solution that fits all. It also shows that there is a need to improve the frequency of text messaging to suit each patient's needs.

Furthermore, some PLWH reported inconvenience while using the adhesive patch. Similar feedback was reported on early versions of the patch with less than 10% of the participants reporting redness and skin itchiness [16, 28]. There is room for improvement in the technology for future developments of the patch. There is also a concern for disclosure of HIV status, as highlighted by the HCPs. Similar concern for unwanted HIV disclosure was reported for Wisepill© and MEMS© [29–31].

For the DMP to function seamlessly, it needs a reliable wireless Internet connection and an electric supply to recharge the iPad/tablet battery. This is something to take into consideration when developing DMP for settings that may not have a reliable network or many power outlets, for the purpose of real-time adherence monitoring. If real-time monitoring is not required but rather timely monitoring the patch can store ingestion data for up to 8 days, so as long as there is connectivity at least once every 8 days, there should be no lost data; once the patient connects to the iPad, all data from the last 8 days will sync automatically. Some PLWH reported an inconvenience of having to be physically close to the tablet while wearing the patch to ensure adequate data transferability. Similar concerns were reported for Wisepill© [26]. This problem may be partly mitigated as the system can utilize cell phones instead of tablets for communicating with the server and this was introduced as a change to the main trial that followed the pilot study. Regarding cost, as most devices were funded as part of a study and were provided free of charge for the participants, we are unable to deduce the cost implications for the patients if they were to purchase those devices themselves or if that would be covered by their health insurance. Finally, the data generated by the DMP can deepen our understanding of individual medication-taking behavior and timing, allowing the development of interventions tailored to each patient's needs.

### 6.2. Strengths and Limitations

The strength of this study is in the triangulation of sources between PLWH experiences and HCPs' opinions, which gives a 360-degree view on a new ARV adherence measuring and monitoring technology. The weaknesses include the small number of participants, mostly male, middle age, MSM (study not generalizable with limited transferability or external validity), and the relatively short duration of using the DMP (4 weeks). However, the main trial of the study with a larger sample size (*n* = 120) and more balanced male-to-female participant ratio is currently ongoing, which will provide rich information on related issues once the trial is completed. Similarly, regarding the sample size of the HCPs, it is relatively small and not representative of the opinions of all HCPs. However, given this is a new technology and not yet widely available for all HCPs and PLWH in a clinical setting, our study provides some initial insights that are of importance. Further research with a bigger sample size would be possible in the future after the widespread of the technology to provide more generalizable results.

## 7. Conclusions

Technology will continue to evolve to advance the ways we can measure medication adherence for research purposes and clinical practice. The DMP is a novel technology of real-time measuring and monitoring of medication adherence with advantages and potentials for improvement. Incorporating changes to the DMP based on the experiences of PLWH and providers will help improve the acceptability of such systems and make it more likely to optimally meet the patients' needs.

## 8. Practical Implications

Analyzing the content of the qualitative interviews from this pilot study guided introducing some changes in the main trial of the DMP that is currently being conducted. For example, using smart phones instead of tablets is now an available option. Future research aims to assess those changes in addition to assessing one's experience with the DMP over a longer time frame.

## Figures and Tables

**Table 1:** Baseline characteristics of PLWH.

Characteristics (*n* = 15)	Mean (SD) or *n* (%)
Age, yrs	50 (6.9)

Gender	
Male	13 (86.7%)

Race and ethnicity	
Black	7 (46.7%)
Hispanic white	6 (40.0%)
Non-Hispanic white	2 (13.3%)

Self-identified major source of HIV infection	
MSM	9 (60.0%)
Heterosexual sex	5 (33.3%)
IV drug use	1 (6.7%)

Duration since HIV diagnosis, yrs	16 (7.0)

Most recent CD4 count, cells/uL (min, max)	774.2 (275, 1375)

Most recent plasma HIV RNA	
Undetectable (<20 copies/mL)	10 (66.7%)
Detectable (≥20 copies/mL)	4 (26.7%)
Unknown	1 (6.7%)

Self-reported missed doses in the past month^*∗*^	
0	3 (20%)
1-2	3 (20%)
>2	5 (33.3%)
Unknown	4 (26.7%)

Missed clinic visits in the past 6 months	
None	8 (53.3%)
1	2 (13.3%)
>1	3 (20%)
Unknown	2 (13.3%)

^*∗*^All patients were on one pill once-daily ARV regimens. SD, standard deviation; MSM, men who have sex with men; HIV, human immunodeficiency virus; IV, intravenous; ARV, antiretroviral; PLWH, people living with HIV.

**Table 2:** Baseline characteristics of HIV healthcare providers.

Characteristics (*n* = 6)	Mean (SD) or *n* (%)
Age, yrs	48 (14)

Gender	
Female	4 (66.6%)

Race and ethnicity	
Asian	3 (50%)
Caucasian/white	1 (16.7%)
Hispanic/Latino	1 (16.7%)
Mixed race	1 (16.7%)

Years of work experience	17 (12.5)

Profession	
Nurse practitioner	4 (66.6%)
Physician	1 (16.6%)
Resident	1 (16.6%)

Number of HIV + patients seen per week	17 (8.2)

Patients' main source of HIV infection	
MSM	5 (83.3%)
Heterosexual sex	1 (16.6%)

Number of patients using DMP	
0	1 (16.6%)
1–5	4 (66.6%
>5	1 (16.6%)

SD, standard deviation; MSM, men who have sex with men; HIV, human immunodeficiency virus; DMP, digital medicine program.

## Data Availability

The data are not available publicly due to the identifying nature of the qualitative interviews.

## Appendix

### Appendix I-a:. Ingestible Sensor Qualitative Interviews

#### Interviewer instructions are given as follows:

Before beginning the audio taping, read the following:

*I would like to talk to you about how you take your medications, and about the Sensor System for taking medications.*

*Your feedback will help us understand what it is like to use the Sensor System. I will start by turning on the audiotape and I will begin to ask you questions.*

*I am very interested in what you have to say. Take as much time as you would like to answer the questions. This is not a test---there are no right or wrong answers. I am interested in hearing how you really feel.*

*Remember, your answers will only be shared with people involved in the study. Nobody else will know what you say. Do you have any questions before we begin?*

#### Medication-taking:

*First, I would like to talk to you about how you take your medication. Tell me about that.*

[*Probes: How do you typically get medication from the pharmacy? Where do you store it? How do you remember to take it?*]

*Tell me about times you have had to change your system for taking medicines.*

[*Probe by asking about the steps the client has described. Then ask about times the system can't work because the pharmacy is closed, person isn't home to take meds with usual routine, forgets a dose, etc.*]

#### Clients' Enrollment into Study:

*Now, I'd like to ask you about how you came to be in this study.*

*Tell me about how you decided to enroll in this study.*

[*Probes: How did you hear about the study? Whose idea was it? Were you pressured to enroll or was it just left up to you?*]

*The Orientation Program:*

*A few days ago, someone from the study explained the Ingestible Sensor System to you. Tell me about that meeting.*

[*Probes: What things did you go over? What parts were helpful? Not so helpful? Were there things you didn't go over that you wish you did?*]

*Now, I'd like to talk to you about the person who explained the Sensor System to you. What's this person like?*

*Tell me your understanding of how the Ingestible Sensor System works.*

[*Probes if not spontaneously mentioned: Tell me about the patch. Tell me about using pills with a special sensor. Tell me about receiving text messages.*]

#### Clients' Overall Experience of the Sensor System:

*Tell me about your experience with the Sensor System. What has it been like for you?*

[*Probes: What did you find helpful about the Sensor System? What was not helpful about the counseling?*]⋯

*What advice would you give to a friend asking whether or not to participate in the Sensor System?*

#### Clients' Experience of Specific Aspects of the Sensor System:

*I'm now going to ask you about specific aspects of the sensor system.*

*Tell me about using the special pills with the sensor in them. What's that like for you?*

*Tell me about wearing the special Sensor System patch. What's that like for you.*

*Tell me about getting text messages. What's that like for you?*

*Tell me about any text messages you received this week.*

[*Probes: Were there messages you found helpful? Were there messages you found not so helpful?*]⋯

*Did you talk to anyone in the Sensor System group? Tell me about it.*

#### Closing:

*Do you recommend any changes in the Sensor System?*

*Thank you very much for sharing your thoughts with me. I appreciate it. Is there anything I haven't asked you about that you want to tell me?*

*Thank you.*

### Weeks 2 and 4 qualitative interview for prepilot phase

#### Omit the following sections:

*Medication-taking:*

*First, I would like to talk to you about how you take your medication. Tell me about that…*

*Clients' Enrollment into Study:*

*Now, I'd like to ask you about how you came to be in this study*⋯

*The Orientation Program:*

*A few days ago, someone from the study explained the Ingestible Sensor System to you. Tell me about that meeting…*

### For weeks 2 and 4 only:

#### After introducing the interview:

*Tell me about the last two weeks using the sensor system.*

### For weeks 2 and 4 only:

#### Before closing the interview:

*Since you started, have you changed how you use the Sensory System?*

*How has using it changed over time? Are there things about it that have been better over time? Worse over time?*

### Appendix I-b

#### Ingestible Sensor Qualitative Interviews for HCP

##### Interviewer Instructions (before beginning the audio taping)**:**

  I will provide a description of the study.
  I am interested in asking your opinion, this will be helpful in the study, I will audio record the conversation. Is this Ok with you? The audio-recording will be anonymous, and your identity will only be revealed to me. Do you agree? If not I will take notes.
  What you share with me will be anonymously published and shared with other people, do you agree?

##### Healthcare providers questions:

  Name:
  Age:
  Profession:
  Years of experience in HIV care:
  Work Setting:
  Education:
  Number of patients seen per week:
  HIV source of infection/patient group (MSM/Drug use/Hetero):
(1) How would you describe the adherence of the patients you see? (probe: Low/moderate/high)
(2) What do you think are the barriers to the adherence?
(3) What do you think are the facilitators to adherence?
(4) Do you know of any adherence aids that can help your patients adhere to their treatment?
(5) Have you heard of the IS? What is your understanding of how it works?
(6) Do you think IS can help your patients with adherence? How? (patch, sensor, text messages)
(7) What issues have your patients been having/do you expect patients will have with IS?
(8) How would IS be different from other adherence real-time monitoring methods?
(9) Do you think IS is a long-term solution or short-term solution to adherence issues? How long do you think patients can be monitored using IS?
(10) Would you recommend IS to your patients? If yes, which patient group? (high/low/moderate adherence) and why?
(11) Would you recommend IS to other HCPs? (If no, why?)
(12) Do you know/heard about IS, what do you think the biggest problem (s) IS may be for using IS technology to monitor HIV patients' adherence?
(13) Is there anything else you would like to add?
